# Enantioselective Evans-Tishchenko Reduction of β-Hydroxyketone Catalyzed by Lithium Binaphtholate

**DOI:** 10.3390/molecules16065008

**Published:** 2011-06-17

**Authors:** Tomonori Ichibakase, Masato Nakatsu, Makoto Nakajima

**Affiliations:** Graduate School of Pharmaceutical Sciences, Kumamoto University, 5-1 Oe-honmachi, Kumamoto 862-0973, Japan

**Keywords:** Evans-Tishchenko reduction, lithium binaphtholate, β-hydroxyketone, 1,3-diol, enantioselectivity

## Abstract

Lithium diphenylbinaphtholate catalyzed the enantioselective Evans-Tishchenko reduction of achiral β-hydroxyketones to afford monoacyl-protected 1,3-diols with high stereoselectivities. In the reaction of racemic β-hydroxyketones, kinetic optical resolution occurred in a highly stereoselective manner.

## 1. Introduction

**Scheme 1:**
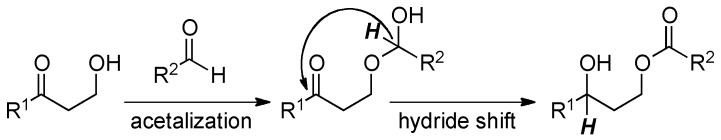
Evans-Tishchenko reduction of β-hydroxyketone.

Stereoselective synthesis of 1,3-diols is an important subject in synthetic organic chemistry because numerous biologically active compounds include such units [1,2]. One method to prepare chiral 1,3-diol is the reduction of β-hydroxyketones, and various metal hydride reagents have been applied to the syntheses of biologically active compounds using this method. Recently, acetalization of a β-hydroxy group with an aldehyde followed by a hydride shift to the carbonyl carbon, the so called Evans-Tishchenko reduction (Scheme 1) [3,4,5], has received much attention because it does not require the use of metal hydride reagents. Although the literature contains numerous examples using the Evans-Tishchenko reduction in the synthesis of biologically active compounds [6,7,8,9,10,11,12,13], the enantioselective Evans-Tishchenko reduction of an achiral β-hydroxyketone had not been reported prior to our preliminary study [14].

We have previously reported that an enantioselective aldol-Tishchenko reaction [14,15,16,17,18,19,20,21] catalyzed by lithium binaphtholate [22,23,24,25,26,27,28,29,30], affords 1,3-diol derivatives from a ketone and an aldehyde. Herein we report an enantioselective Evans-Tishchenko reduction, which yields optically active mono-acyl protected 1,3-diols from the reaction of achiral β-hydroxyketones with aldehydes catalyzed by lithium binaphtholate.

## 2. Results and Discussion

### 2.1. Optimization of Reaction Conditions

First we investigated the Evans-Tishchenko reduction of α,α-dimethyl-β-hydroxypropiophenone (**2a**) with benzaldehyde (**3a**) in THF at r.t. using lithium binaphtholate **1a**, prepared *in situ* from binaphthol and BuLi, as a catalyst (Table 1, entry 1). The reaction gave the corresponding monobenzoyl 1,3-diol **4aa**, but with low chemical yield (36%) and enantioselectivity (2% *ee*). Screening binaphthol derivatives revealed that introducing substituents to the 3,3’-positions of the catalyst dramatically increased both the chemical yield and enantioselectivity (entries 2–6). Among the various substituents surveyed, phenyl groups gave the best result (entry 4, 82% yield, 96% *ee*). Compared to THF, the use of ether or toluene as solvent gave unsatisfactory results (entries 7 and 8). Although lowering the reaction temperature increased the enantioselectivity (entry 9), the reaction did not proceeded smoothly at −78 °C (entry 10).

**Table 1:** Evans-Tishchenko reduction of α,α-dimethyl-β-hydroxypropiophenone. 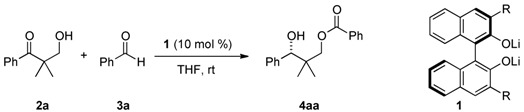

Entry	Catalyst	Conditions	Solvent	Yield, % ^a^	*ee*, % ^b^
1	**1a** (R = H)	rt, 24 h	THF	36	2
2	**1b** (R = Me)	rt, 24 h	THF	91	79
3	**1c** (R = Br)	rt, 24 h	THF	76	83
4	**1d** (R = Ph)	rt, 0.5 h	THF	82	96
5	**1e** (R = 4-MeC_6_H_4_)	rt, 0.5 h	THF	80	89
6	**1f** (R = 3,5-Me_2_C_6_H_3_)	rt, 0.5 h	THF	84	20
7	**1d** (R = Ph)	rt, 0.5 h	Et_2_O	73	56
8	**1d** (R = Ph)	rt, 0.5 h	toluene	63	63
9	**1d** (R = Ph)	−40 °C, 0.5 h	THF	87	99
10	**1d** (R = Ph)	−78 °C, 48 h	THF	56	99

^a^ Isolated yield; ^b^ Determined by HPLC analysis.

### 2.2. Evans-Tishchenko Reduction of Various Achiral β-Hydroxy ketones

With the optimum conditions in hand, we next examined the Evans-Tishchenko reduction of various β-hydroxyketones and aldehydes. The reaction of **2a** with pivalaldehyde (**3b**) as a hydride source, which should afford pivaloyl ester, gave the corresponding product **4ab** with the same absolute configuration as that with benzaldehyde in high yield at 0 °C, but was accompanied by the side product **5ab** (5% yield), which was formed by transesterification of **4ab** (Table 2, entry 2). The obtained enantioselectivity decreased to 90% *ee*, probably due to the higher reaction temperature (0 °C in entry 2 *vs*. −40 °C in entry 1). β-Hydroxyketone **2b**, with cyclohexane at the α-position gave similar results as **2a** with benzaldehyde (**3a**) or pivaldehyde (**3b**) (entries 3 and 4). Isopropyl ketone **2c** gave the product **4ca** in high enantioselectivity, but accompanied by 31% of the transesterification product **5ca** (entry 5). Under the reaction conditions, either **4ca** or **5ca** was isomerized into a mixture of **4ca** and **5ca** (**4ca**:**5ca** = 2:1) without losing the enantioselectivity. The reaction of methyl ketone **2d** and benzaldehyde (**3a**) gave excellent results, however, the products **4da** and **5da** (**4da**:**5da** = 2:1) were isolated as a single dibenzoyl ester **7da** after benzoylation of the mixture of **4da** and **5da** (entry 6), because the monobenzoyl products **4da** and **5da** could not be separated.

**Table 2:** Evans-Tishchenko reduction of various achiral β-hydroxy ketones. 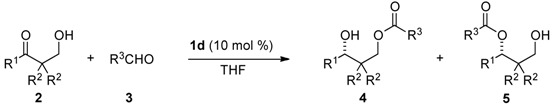

Entry	2	3	Conditions	4	Yield of 4 (5) % ^a^	*ee* of 4 (5) % ^b^
1	**2a**	**3a**	−40 °C, 0.5 h	**4aa**	87 (0)	99
2	**2a**	**3b**	0 °C, 1 h	**4ab**	93 (5)	90 (90)
3	**2b**	**3a**	−40 °C, 0.5 h	**4ba**	96 (0)	98
4	**2b**	**3b**	0 °C, 4 h	**4bb**	82 (13)	83
5	**2c**	**3a**	rt, 4 h	**4ca**	64 (31)	93 (93)
6	**2d**	**3a**	−40 °C, 6 h	**4da**	91 ^c^ [**4da**:**5da **= 2:1]	99 ^c^

^a^ Isolated yield; ^b^ Determined by HPLC analysis; ^c^ Isolated as dibenzoyl ester **7da**. Ratio of **4da** to **5da** was calculated from the crude NMR before benzoylation.
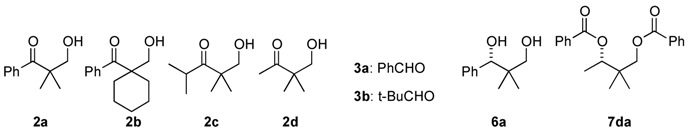

### 2.3. Evans-Tishchenko Reduction of Chiral β-Hydroxypropiophenone

**Scheme 2:**
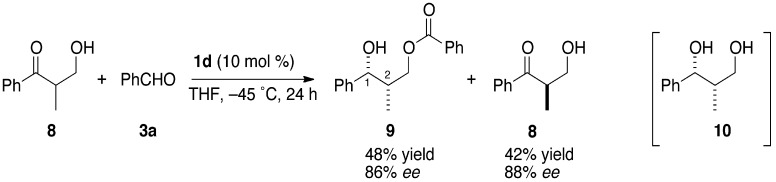
Kinetic resolution of chiral β-hydroxyketone.

**Scheme 3:**
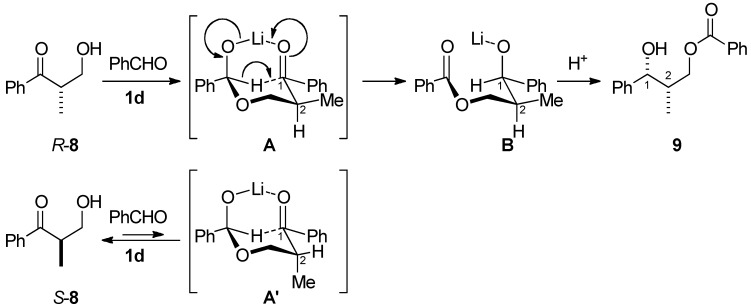
Plausible reaction pathway.

Using chiral β-hydroxyketones under the above conditions, a kinetic optical resolution occurred in a highly enantioselective manner (Scheme 2). The reaction of racemic α-methyl-β-hydroxyketone **8** and benzaldehyde (**3a**) at −45 °C for 24 h afforded monobenzoyl 1,3-diol **9** in 48% yield with 86% *ee*, and the unreacted starting material **8** was recovered in 42% yield with 88% *ee*. The stereochemistry of **9** was determined by the conversion to the stereochemically-known diol **10** [31] by debenzoylation with sodium methoxide in methanol. Scheme 3 explains the 1,2-*syn* stereochemistry using bicyclic transition model **A** for the reaction of **8** and benzaldehyde **3a**. The α-methyl group preferred the equatorial position over the axial position, producing 1,2-*syn* product predominantly.

### 2.4. Reaction of An α-Unsubstituted-β-Hydroxypropiophenone

**Scheme 4:**
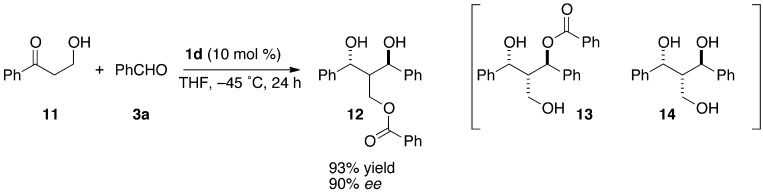
The reaction of an α*-*unsubstituted*-*β*-*hydroxyketone.

In the reaction of α-unsubstituted-β-hydroxypropiophenone **11**, the Evans-Tishchenko product was not observed, but rather the monobenzoyl triol **12** was obtained in high yield with a high enantioselectivity (Scheme 4). Compound **12** may be formed by the transesterification of the aldol-Tishchenko adduct **13**. It is interesting that the Evans-Tishchenko reduction proceeded exclusively in methyl ketone **2d**, while β-hydroxypropiophenone (**11**) gave predominantly the aldol-Tishchenko adduct, although both hydroxyketones have enolizable positions. This may be because the Li-coordinated cyclic structure promoted the enolate formation or the approach of the aldehyde, though the detail is not clear. The absolute configuration of **12** was determined by conversion to stereochemically-known triol **14** [32].

## 3. Experimental

### 3.1. General

^1^H-NMR and ^13^C-NMR spectra were recorded using a JEOL JNM-ECX-400 (^1^H, 400 MHz; ^13^C, 100 MHz) spectrometer. Chemical shift values are expressed in ppm relative to internal tetramethylsilane. Coupling constants (*J*) are reported in Hz. Abbreviations are as follows: s, singlet; d, doublet; t, triplet; q, quartet; m, multiplet; br, broad. Infrared spectra were recorded using a JASCO FT/IR-5300. HPLC was performed on a JASCO PU-1580 with a JASCO UV-1575 (λ = 254 nm), and chiral separations were performed using Daicel Chiralpak or Chiralcel columns (ϕ 0.46 × 25 cm) with mixtures of hexane/isopropyl alcohol (hex/IPA) as eluents. Optical rotations were obtained using a JASCO DIP-370 digital polarimeter. β-Hydroxyketones **2**, **6** and **8** were prepared according to the literature [33,34,35,36], as were the binaphthol derivatives [37].

### 3.2. Enantioselective Evans-Tishchenko Reduction of β-Hydroxyketones

#### 3.2.1. (*S*)-2,2-Dimethyl-1-phenyl-1,3-propanediol 3-*O*-benzoate (**4aa**) [38]

Under an argon atmosphere, *n*-BuLi (0.094 mmol, 20 mol %) in hexane (0.17 M, 0.55 mL) was added to a solution of (*R*)-3,3’-diphenylbinaphthol (21 mg, 0.047 mmol, 10 mol %) in THF at −45 °C, and the mixture was stirred for 5 min. Then solutions of benzaldehyde (**3a**, 75 mg, 0.708 mmol, 1.5 equiv.) and 3-hydroxy-2,2-dimethyl-1-phenylpropan-1-one (**2a**, 84 mg, 0.472 mmol, 1.0 equiv.) were successively added to the above mixture. After 0.5 h, the reaction was quenched with sat. NH_4_Cl aq. and the mixture was stirred for an additional 10 min at r.t. The aqueous layer was extracted with ethyl acetate and the combined organic layer was washed with brine. After drying over Na_2_SO_4_ and evaporating the solvent, the residue was purified by silica gel column chromatography (dichloromethane) to afford diol derivative **4aa** (114 mg, 87% yield, 99% *ee*) as colorless prisms. Mp 73–74 °C. ^1^H-NMR (CDCl_3_): δ 0.97 (s, 3H, C*H*_3_), 1.04 (s, 3H, C*H*_3_), 2.45 (brs, 1H, –O*H*), 4.02 (d, 1H, *J* = 11.0 Hz, OC*H*_2_), 4.43 (d, 1H, *J* = 11.0 Hz, OC*H*_2_), 4.69 (s, 1H, C*H*Ph), 7.25–7.48 (m, 7H, Ar*H*), 7.56–7.60 (m, 1H, Ar*H*), 8.04–8.06 (m, 2H, Ar*H*). HPLC (Daicel Chiralpak AD-H, hex/IPA = 9/1, 1.0 mL/min): *t*_R_ 8.8 (*S*), 12.8 min (*R*). [α]^30^_D_−23.1 (*c* 1.13, CHCl_3_) for 99% *ee*.

#### Determination of the absolute configuration of **4aa**.

To a solution of **4aa** (114 mg, 0.401 mmol, 1.0 equiv.) in MeOH (2 mL), NaOMe (0.05 mmol, 12 mol %) in MeOH (0.1 mL) was added and the resulting homogeneous mixture was stirred for 3 h. The mixture was diluted with ethyl acetate (20 mL), and washed with water (5 mL). The aqueous layer was extracted twice with ethyl acetate (10 mL × 2). The combined organic layers were washed with brine (10 mL) and dried over Na_2_SO_4_. After concentration *in vacuo*, the residue was purified by silica gel column chromatography (hexane/ethyl acetate = 4/1) to give diol **6a** (70 mg, 96% yield, 99% *ee*) as colorless needles. The optical rotation data shows (+)-**6a** had an *S*-configuration, indicating (−)-**4aa** has an *S*-configuration. Mp 62–63 °C. ^1^H-NMR (CDCl_3_) δ 0.79 (s, 3H, C*H*_3_), 0.84 (s, 3H, C*H*_3_), 3.43 (d, 1H, *J =* 10.6 Hz, OC*H*_2_), 3.50–3.58 (m, 2H, OC*H*_2_ and C*H*Ph), 3.77 (brs, 1H, O*H*), 4.57 (s, 1H, O*H*), 7.32–7.35 (m, 5H, Ar*H*). [α]^30^_D_ +44.7 (*c* 1.00, CHCl_3_) for 99% *ee* (*S*). [lit. 39: [α]_D_^30^ +21.7 (*c* 1.17, CHCl_3_) for 55% *ee* (*S*)].

#### 3.2.2. (*S*)-2,2-Dimethyl-1-phenyl-1,3-propanediol 3-*O*-pivaloate (**4ab**)

^1^H-NMR (CDCl_3_): δ 0.87 (s, 3H, C(C*H*_3_)_2_), 0.95 (s, 3H, C(C*H*_3_)_2_), 1.26 (s, 9H, C(C*H*_3_)_3_), 2.39 (d, 1H, *J* = 3.2 Hz, –O*H*), 3.74 (d, 1H, *J* = 11 Hz, C*H*_2_), 4.19 (d, 1H, *J* = 11 Hz, C*H*_2_), 4.57 (d, 1H, *J* = 3.2 Hz, PhC*H*), 7.26–7.33 (m, 5H, Ar*H*). ^13^C-NMR: δ 19.4, 21.3, 27.2, 39.0, 39.3, 70.5, 78.0, 127.5, 127.6, 127.7, 140.9, 178.6. IR (CHCl_3_): 3480, 1715 cm^−1^. MS (FAB): *m/z* 173, 287 ([M+Na]^+^). HRMS: calcd for C_16_H_24_O_3_Na 287.1623, found 287.1619. HPLC (Daicel Chiralcel OD-H, hex/IPA = 9/1, 1.0 mL/min) *t*_R_: 5.1 (*R*), 6.7 (*S*) min. [α]^17^_D_ −7.5 (*c* 1.0, CHCl_3_) for 90% *ee* (*S*). The absolute configuration of **4ab** was determined by conversion to diol **6a**. [α]^21^_D_ +35.1 (*c* 1.37, CHCl_3_) for 90% *ee* (*S*). [lit. 39: [α]^30^_D_ +21.7 (*c* 1.17, CHCl_3_) for 55% *ee* (*S*)].

#### 3.2.3. (*S*)-2,2-Dimethyl-1-phenyl-1,3-propanediol 1-*O*-pivaloate (**5ab**)

^1^H-NMR (CDCl_3_): δ 0.88 (s, 3H, C(C*H*_3_)_2_), 0.89 (s, 3H, C(C*H*_3_)_2_), 1.25 (s, 9H, C(C*H*_3_)_3_), 2.34 (brs, 1H, O*H*), 3.24 (d, 1H, *J* = 11 Hz, C*H*_2_), 3.42 (d, 1H, *J* = 11 Hz, C*H*_2_), 5.77 (s, 1H, PhC*H*), 7.28–7.35 (m, 5H, Ar*H*). ^13^C-NMR: δ 19.2, 21.6, 27.2, 39.0, 40.2, 69.1, 78.5, 127.66, 127.72, 127.8, 137.5, 178.2. IR (neat): 3458, 1730 cm^−1^. MS (FAB, NBA+NaI): *m/z* 287 ([M+Na]^+^). HRMS: calcd for C_16_H_24_O_3_Na 287.1623, found 287.1628. [α]^17^_D_ +51.4 (*c* 0.6, CHCl_3_). 

#### 3.2.4. (−)-2,2-Pentamethylene-1-phenyl-1,3-propanediol 3-*O*-benzoate (**4ba**)

^1^H-NMR (CDCl_3_): δ 1.17–1.26 (m, 1H, CC*H*_2_C), 1.37–1.61 (m, 9H, CC*H*_2_C), 2.50 (brs, 1H, O*H*), 4.21 (d, 1H, *J* = 11.4 Hz, OC*H*_2_), 4.47 (d, 1H, *J* = 11.4 Hz, OC*H*_2_), 4.74 (s, 1H, PhC*H*), 7.21–7.32 (m, 5H, Ar*H*), 7.41–7.44 (m, 2H, Ar*H*), 7.54–7.58 (m, 1H, Ar*H*), 7.94–7.96 (m, 2H, Ar*H*). ^13^C-NMR: δ 21.3, 21.4, 25.8, 28.2, 28.3, 41.3, 65.2, 78.2, 127.3, 127.6, 127.7, 128.3, 129.5, 130.1, 132.9, 141.0, 166.4. IR (neat): 3502, 1716 cm^−1^. MS (FAB, NBA+NaI): *m/z* 347 ([M+Na]^+^). HRMS: calcd for C_21_H_24_O_3_Na 347.1623, found 347.1643. HPLC (Daicel Chiralpak AD-H, hex/IPA = 49/1, 1 mL/min)*t*_R_: 13.0 (major), 14.4 (minor) min. [α]^18^_D_ −11.4 (*c* 1.13, CHCl_3_) for 98% *ee*.

#### 3.2.5. (+)-2,2-Pentamethylene-1-phenyl-1,3-propanediol 3-*O*-pivaloate (**4bb**)

^1^H-NMR (CDCl_3_): δ 1.22 (s, 9H, C(CH_3_)_3_), 1.34-1.58 (m, 10H, CC*H*_2_C), 2.54 (brs, 1H, O*H*), 3.94 (d, 1H, *J* = 11.4 Hz, OC*H*_2_), 4.21 (d, 1H, *J* = 11.4 Hz, OC*H*_2_), 4.62 (s, 1H, PhC*H*), 7.25–7.32 (m, 5H, Ar*H*). ^13^C-NMR: δ 21.2, 21.4, 25.8, 27.2, 28.2, 28.3, 38.9, 41.1, 64.9, 78.5, 127.3, 127.6, 127.7, 140.9, 178.3. IR (KBr): 3498, 1711 cm^−1^. MS (FAB, NBA+NaI): *m/z* 287, 327 ([M+Na]^+^). HRMS: calcd for C_19_H_28_O_3_Na 327.1936, found 327.1929. HPLC (Daicel Chiralpak AD-H, hex/IPA = 49/1, 1 mL/min)*t*_R_: 28.1 (minor), 33.0 (major) min. [α]^17^_D_ +6.0 (*c* 1.36, CHCl_3_) for 83% *ee*.

#### 3.2.6. (+)-2,2-Pentamethylene-1-phenyl-1,3-propanediol 1-*O*-pivaloate (**5bb**)

^1^H-NMR (CDCl_3_): δ 1.12-1.59 (m, 9H, CC*H*_2_C), 1.24 (s, 9H, C(C*H*_3_)_3_), 1.69–1.72 (m, 1H, CC*H*_2_C), 2.07 (brs, 1H, O*H*), 3.49 (d, 1H, *J* = 11.9 Hz, OC*H*_2_), 3.63 (d, 1H, *J* = 11.9 Hz, OC*H*_2_), 5.78 (s, 1H, PhC*H*), 7.26–7.33 (m, 5H, Ar*H*). ^13^C-NMR: δ 21.1, 21.3, 25.9, 27.2, 28.2, 28.4, 39.0, 41.9, 62.9, 79.8, 127.7, 127.8, 127.9, 137.3, 177.6. IR (neat): 3513, 1732 cm^−1^. MS (FAB, NBA+NaI): *m/z* 57, 91, 305 ([M+H]^+^). HRMS: calcd for C_19_H_29_O_3_ 305.2117, found 305.2128. [α]^17^_D_ +23.3 (*c* 1.47, CHCl_3_).

#### 3.2.7. (*S*)-2,2,4-Trimethyl-1,3-pentanediol 1-*O*-benzoate (**4ca**)

^1^H-NMR (CDCl_3_): δ 0.97 (d, 3H, *J* = 6.9 Hz, CH(C*H*_3_)_2_), 1.02 (d, 3H, *J* = 6.9 Hz, CH(C*H*_3_)_2_), 1.05 (s, 3H, C(C*H*_3_)_2_), 1.07 (s, 3H, C(C*H*_3_)_2_), 1.97–2.00 (m, 2H, O*H*, C*H*(CH_3_)_2_), 3.38 (s, 1H, C*H*OH), 4.02 (d, 1H, *J* = 11 Hz, C*H*_2_), 4.38 (d, 1H, *J* = 11 Hz, C*H*_2_), 7.43–7.47 (m, 2H, Ar*H*), 7.55–7.59 (m, 1H, Ar*H*), 8.03–8.05 (m, 2H, Ar*H*). ^13^C-NMR: δ 17.9, 19.7, 22.3, 23.0, 28.6, 40.3, 70.0, 80.3, 128.5, 129.8, 129.9, 133.2, 167.6. IR (KBr): 3340, 1724 cm^−1^. MS (FAB, NBA+NaI): *m/z* 77, 105, 273 ([M+Na]^+^). HRMS calcd for C_15_H_22_O_3_Na 273.1467, found 273.1458. HPLC (Daicel Chiralpak AD-H, hex/IPA = 39/1, 1.0 mL/min) *t*_R_: 14.4 (*S*), 15.7 (*R*) min. [α]^17^_D_ +9.5 (*c* 2.14, benzene) for 93% *ee*, (*S*). The absolute configuration of **4ca** was determined by conversion to diol **6a**. [α]^29^_D_ −11.3 (*c* 1.54, CH_2_Cl_2_) for 93% *ee*. [lit. 40: [α]^30^_D_ −9.5 (*c* 1.0, CH_2_Cl_2_) for 75% *ee*, (*S*)].

#### 3.2.8. (*S*)-2,2,4-Trimethyl-1,3-pentanediol 3-*O*-benzoate (**5ca**)

^1^H-NMR (CDCl_3_): δ 0.95 (s, 3H, C(C*H*_3_)_2_), 1.01 (d, 3H, *J* = 6.9 Hz, CH(C*H*_3_)_2_), 1.09 (d, 3H, *J* = 6.9 Hz, CH(C*H*_3_)_2_), 1.10 (s, 3H, C(C*H*_3_)_2_), 2.17–2.21 (m, 1H, C*H*(CH_3_)_2_), 2.79 (brs, 1H, O*H*), 3.09 (d, 1H, *J* = 11.9 Hz, C*H*_2_), 3.34 (d, 1H, *J* = 11.9 Hz, C*H*_2_), 5.04 (d, 1H, *J* = 2.8 Hz, OC*H*), 7.45–7.49 (m, 2H, Ar*H*), 7.58–7.61 (m, 1H, Ar*H*), 8.08–8.10 (d, 2H, *J* = 7.3 Hz, Ar*H*). ^13^C-NMR: δ 17.9, 19.7, 22.3, 23.0, 28.6, 40.3, 70.0, 80.3, 128.5, 129.8, 129.9, 133.2, 167.6. IR (neat): 3493, 1720 cm^−1^. MS (FAB, NBA+NaI): *m/z* 105, 273 ([M+Na]^+^). HRMS：calcd for C_15_H_22_O_3_Na 273.1467, found 273.1447. HPLC (Daicel Chiralpak AD-H, hex/IPA = 39/1, 1 mL/min) *t*_R_: 13.5 (*S*), 14.8 (*R*) min. [α]^17^_D_ −15.4 (*c* 1.15, CHCl_3_) for 93% *ee*, (*S*).

Treatment of **4ca** (93% *ee*) with 10 mol % of **1d** for 3 h at rt afforded **5ca** (32% yield, 92% *ee*) with unreacted **4ca** (63% yield, 93% *ee*). Treatment of **5ca** (93% *ee*) with 10 mol % of **1d** for 3 h at r.t. afforded **4ca** (58% yield, 93% *ee*) with unreacted **5ca** (32% yield, 93% *ee*).

#### 3.2.9. (+)-2,2-Dimethyl-1,3-butanediol dibenzoate (**7da**)

Starting from **2d** (0.057 mL, 0.470 mmol, 1.0 equiv.) and **3a**(75 mg, 0.708 mmol, 1.5 equiv.), a mixture of **4da** and **5da** was obtained by the above method. To the solution of the mixture in dichloromethane (2 mL), benzoyl chloride (0.820 mL, 0.710 mmol, 1.5 equiv.), Et_3_N (0.147 mL, 1.41 mmol, 3.0 equiv.) and DMAP (11.5 mg, 0.094 mmol, 10 mol %) were added successively. After being stirred for 12 h at r.t., the reaction mixture was quenched by diethylamine (0.1 mL) and was diluted with ethyl acetate. The organic layer were washed with HCl aq., NaHCO_3_ aq. and brine (10 mL) successively, and dried over Na_2_SO_4_. After concentration *in vacuo*, the residue was purified by silica gel column chromatography (hexane/dichloromethane = 1/1) to give dibenzoate **7da **(141 mg, 92% yield, 99% *ee*) as as oil. ^1^H-NMR (CDCl_3_) δ 1.13 (s, 3H, CC*H*_3_), 1.17 (s, 3H, CC*H*_3_), 1.37 (d, 3H, *J* = 6.4 Hz, CHC*H*_3_), 4.22 (d, 1H, *J* = 11.5 Hz, C*H*_2_), 4.26 (d, 1H, *J* = 11.5 Hz, C*H*_2_), 5.28 (q, 1H, *J* = 6.4 Hz, C*H*), 7.39–7.44 (m, 4H, Ar*H*), 7.51–7.55 (m, 2H, Ar*H*), 8.03–8.06 (m, 4H, Ar*H*). ^13^C-NMR (CDCl_3_) δ 14.70, 20.38, 21.33, 38.07, 70.02, 74.50, 128.24, 128.28, 129.39, 129.45, 130.02, 130.42, 132.76, 132.83, 165.66, 166.28. IR (neat): 1720 cm^−1^. MS (FAB, NBA+NaI) 327 ((M+H)^+^), 205, 154, 137, 105, 83, 77. HRMS (FAB) calcd for C_20_H_23_O_4_ ((M+H)^+^) 327.1602, found 327.1597. [α]^29^_D_ +66.6 (*c* 1.47, CHCl_3_) for 99% *ee*. HPLC (Daicel Chiralpak AD-H, hex/IPA = 200/1, 1.0 mL/min): *t*_R_: 19.3 (minor), 20.7 (major) min.

### 3.3. Kinetic Optical Resolution of 3-Hydroxy-2-methyl-1-phenylpropan-1-one (**8**)

Under an argon atmosphere, *n*-BuLi (0.094 mmol, 20 mol %) in hexane (0.19 M, 0.49 mL) was added to a solution of (*R*)-3,3’-diphenylbinaphthol (21 mg, 0.047 mmol, 10 mol %) in THF at −45 °C, and the mixture was stirred for 5 min. Then solutions of benzaldehyde (**3a**, 50 mg, 0.470 mmol, 1.0 equiv.) and racemic 3-hydroxy-2-methyl-1-phenylpropan-1-one (**8**, 77 mg, 0.470 mmol, 1.0 equiv.) were successively added to the above mixture. After 24 h, the reaction was quenched with sat. NH_4_Cl aq. and the mixture was stirred for 20 min at r.t. The aqueous layer was extracted twice with dichloromethane and the combined organic layers were washed with brine. After drying over Na_2_SO_4_ and evaporating the solvent, the residue was purified by silica gel column chromatography (hexane/ethyl acetate = 6/1 ~ 2/1) to afford the diol derivative **9** (61 mg, 48% yield, 86% *ee*) and unreacted starting material **8** (32 mg, 42%, 88% *ee*).

#### 3.3.1. (*1R*,*2S*)-2-Methyl-1-phenyl-1,3-propanediol 3-*O*-benzoate (**9**)

^1^H-NMR (CDCl_3_): δ 1.03 (d, 3H, *J* = 6.9 Hz, C*H*_3_), 2.16 (dd, 1H, *J* = 1.4, 3.6 Hz, O*H*), 2.28–2.36 (m, 1H, C*H*CH_3_), 4.14 (dd, 1H, *J* = 6.0, 11 Hz, C*H*_2_), 4.43 (dd, 1H, *J* = 6.7, 11 Hz, C*H*_2_), 4.84–4.86 (m, 1H, PhC*H*), 7.26–2.29 (m, 1H, Ar*H*), 7.34–7.36 (m, 4H, Ar*H*), 7.43−7.47 (m, 2H, Ar*H*), 7.56–7.59 (m, 1H, Ar*H*), 8.01−8.03 (m, 2H, Ar*H*). ^13^C-NMR: δ 11.2, 40.1, 67.1, 74.3, 126.0, 127.4, 128.3, 128.4, 130.0, 130.1, 133.0, 142.7, 166.7. IR (neat): 3492, 1722 cm^−1^. MS (FAB, NBA+NaI): *m/z* 77, 105, 154, 271 ([M+H]^+^). HRMS: calcd for C_17_H_19_O_30_ 271.1334 found 271.1356. HPLC (Daicel Chiralpak AD-H, hex/IPA = 9/1, 1.0 mL/min) *t*_R_: 9.8 (1*R*,2*S*), 11.2 (1*S*,2*R*) min. [α]^18^_D_ −17.4 (*c* 1.45, CHCl_3_) for 88% *ee*, (1*R*,2*S*). The relative and absolute configuration of **9** were determined by conversion to diol **10**. [α]^21^_D_ +39.2 (*c* 0.88, CHCl_3_) for 88% *ee* (1*R*,2*S*). [lit. 31: [α]^27^_D_ +56.1 (*c* 0.75, CHCl_3_) for >99% *ee*, (1*R*,2*S*)].

#### 3.3.2. (*S*)-3-Hydroxy-2-methyl-1-phenylpropan-1-one (**8**)

HPLC (Daicel Chiralpak AD-H, hex/IPA = 19/1, 1 mL/min) *t*_R_: 17.6 (*R*), 19.7 (*S*) min. [α]^29^_D_ −37.9 (*c* 0.86, EtOH) for 86% *ee* (*S*). [lit. 41: [α]^23^_D_ +41.2 (*c* 0.90, EtOH) for 91% *ee*, (*R*)].

### 3.4. The Aldol-Tishchenko Reaction of An α-Unsubstituted-β-Hydroxypropiophenone

#### (1R,3R)-2-Benzoyloxymethyl-1,3-diphenyl-1,3-propanediol (**12**)

Under an argon atmosphere, *n*-BuLi (0.094 mmol, 20 mol %) in hexane (0.19 M, 0.49 mL) was added to a solution of (*R*)-3,3’-diphenylbinaphthol (21 mg, 0.047 mmol, 10 mol %) in THF at −45 °C, and the mixture was stirred for 5 min. Then solutions of benzaldehyde (**3a**, 76 mg, 0.716 mmol, 1.5 equiv.) and 3-hydroxy-1-phenylpropan-1-one (**11**, 70 mg, 0.470 mmol, 1.0 equiv.) were successively added to the above mixture. After 24 h, the reaction was quenched with sat. NH_4_Cl aq. and the mixture was stirred for 20 min at r.t. The aqueous layer was extracted twice with dichloromethane and the combined organic layers were washed with brine. After drying over Na_2_SO_4_ and evaporating the solvent, the residue was purified by silica gel column chromatography (hexane/ethyl acetate = 1/1 then dichloromethane) to afford the diol derivative **12** (158 mg, 93% yield, 90% *ee*) as colorless needles. Mp 119–120 °C. ^1^H-NMR (CDCl_3_): δ 2.45–2.50 (m, 1H, CC*H*C), 3.89 (d, 1H, *J* = 6.7 Hz, O*H*), 3.90 (d, 1H, *J* = 4.6 Hz, O*H*), 4.20 (dd, 1H, *J* = 5.5, 11.5 Hz, BzOC*H*_2_), 4.65 (dd, 1H, *J* = 8.3, 11.5 Hz, BzOC*H*_2_), 5.33–5.57 (m, 1H, HOC*H*), 5.11 (dd, 1H, *J* = 4.6, 4.6 Hz, HOC*H*), 7.16–7.20 (m, 3H, Ar*H*), 7.24–7.30 (m, 3H, Ar*H*), 7.35–7.43 (m, 6H, Ar*H*), 7.50–7.54 (m, 1H, Ar*H*), 7.80–7.83 (m, 2H, Ar*H*). ^13^C-NMR: δ 50.7, 61.7, 71.6, 73.5, 125.4, 125.7, 127.0, 127.6, 128.2, 128.6, 129.5, 129.7, 133.0, 141.9, 142.2, 166.8. IR (neat): 3477, 1713 cm^−1^. MS (FAB, NBA+NaI) 385 ((M+Na)^+^), 326, 323, 242, 176, 173, 92, 77. HRMS (FAB) calcd for C_23_H_22_O_4_Na ((M+Na)^+^) 385.1420, found 385.1416. HPLC (Daicel Chiralpak AD-H, hex/IPA = 9/1, 1.0 mL/min) *t*_R_: 19.3 (*S,S*), 25.1 (*R,R*) min. [α]^29^_D_ +44.1 (*c* 1.04, CHCl_3_) for 92% *ee*, (*S,S*).

The absolute configuration of **12** was determined by the conversion to triol **14**. ^1^H-NMR (CDCl_3_) δ 1.91 (brs, 1H, O*H*), 3.25 (brs, 1H, *J* = 10.6 Hz, C*H*_2_), 3.65 (dd, 1H, *J* = 5.48, 11.44 Hz, C*H*), 4.18 (brs, 1H, O*H*), 4.43 (d, 1H, *J* = 4.12 Hz, C*H*_2_), 7.16–7.19 (m, 3H, Ar*H*), 7.22–7.25 (m, 3H, Ar*H*), 7.27–7.33 (m, 3H, Ar*H*). [α]^18^_D_ −35.0 (*c* 2.16, CH_2_Cl_2_) for 92% *ee* [lit. 32: [α]^20^_D_ −39 (*c* 0.97, CH_2_Cl_2_) for >99% *ee*, (*R,R*)].

## 4. Conclusions

We have demonstrated that lithium diphenylbinaphtholate catalyzes the enantioselective Evans-Tishchenko reduction of β-hydroxyketones, affording monoacyl-protected 1,3-diols in high stereoselectivities. In the reaction of racemic β-hydroxyketone, kinetic optical resolution occurs in a highly stereoselective manner. Further investigations to expand the substrate scope and to explore the reaction mechanism are currently underway.
